# Polymer Screening for Efficient Water Cut Reduction in a Sandstone Oilfield in Kazakhstan

**DOI:** 10.3390/polym15081969

**Published:** 2023-04-21

**Authors:** Daniyar Yerniyazov, Madi Yesmukhambet, Razida Kenes, Azamat Bukayev, Mariam Shakeel, Peyman Pourafshary, Darya Musharova

**Affiliations:** 1 School of Mining and Geosciences, Nazarbayev University, Astana 010000, Kazakhstan; 2 KazMunayGas Engineering, Astana Z05H9E8, Kazakhstan

**Keywords:** polymer flooding, rheology, thermal stability, HPAM, adsorption

## Abstract

Polymer flooding is one of the most widely used and effective enhanced oil recovery techniques. It can improve the macroscopic sweep efficiency of a reservoir by controlling the fractional flow of water. The applicability of polymer flooding for one of the sandstone fields in Kazakhstan was evaluated in this study and polymer screening was carried out to choose the most appropriate polymer among four hydrolyzed polyacrylamide polymer samples. Polymer samples were prepared in Caspian seawater (CSW) and assessed based on rheology, thermal stability, sensitivity to non-ionic materials and oxygen, and static adsorption. All the tests were performed at a reservoir temperature of 63 °C. Based on the results of the screening study, tolerance of a polymer towards high-temperature reservoir conditions, resistance to bacterial activity and dissolved oxygen present in make-up brine, chemical degradation, and reduced adsorption on rock surface were considered the most important screening parameters. As a result of this screening study, one out of four polymers was selected for the target field as it showed a negligible effect of bacterial activity on thermal stability. The results of static adsorption also showed 13–14% lower adsorption of the selected polymer compared to other polymers tested in the study. The results of this study demonstrate important screening criteria to be followed during polymer selection for an oilfield as the polymer should be selected based on not only polymer characteristics but also the polymer interactions with the ionic and non-ionic components of the make-up brine.

## 1. Introduction

When the primary drive mechanism is insufficient to maintain optimum reservoir pressure to produce oil, the secondary drive mechanism is applied by injecting gas or water. Waterflooding can increase oil recovery by 10% to 30%. The effectiveness of the waterflooding process highly depends on the characteristics of the field, such as rock/fluid properties and mobility ratio, as shown in Equation (1):

(1) $$M=\frac{\frac{k_{rw}}{\mu_{w}}}{\frac{k_{ro}}{\mu_{o}}}$$

where *M* is the mobility ratio, *k_rw_*, and *k_ro_* are water and oil relative permeabilities, respectively, and *μ_w_* and *μ_o_* are water and oil viscosities, respectively.

Mobility is critical to evaluate the waterflooding performance. The higher the water–oil mobility ratio (M), the more challenges are observed. At M values greater than one, early water breakthrough, rapid decline in oil production, and low recovery were observed [1,2,3].

Additionally, besides the poor recovery factor, a widespread high water cut problem can occur. Depending on the initial reservoir conditions such as initial water saturation in the reservoir or production strategies before water flooding, a high water cut can become a severe problem in the field. Once water breakthrough occurs, the injected water flows through already existing least-resistant water paths, bypassing untouched oil regions [4]. Hence, it is necessary to control and reduce the water cut by altering the water macroscopic sweep efficiency. To prevent such problems, enhanced oil recovery (EOR) methods such as polymer flooding can be applied.

Polymer flooding is a well-established chemical EOR technique that aids in overcoming mobility-related issues. Polymer flooding helps to reach the residual oil saturation faster and even in a more economically feasible manner. It mainly affects macroscopic sweep efficiency [5]. By decreasing the mobility ratio and covering a larger reservoir area with injected fluid, polymer flooding yields better results by efficiently recovering the inaccessible oil in the reservoir [6]. During laboratory experiments performed by Alfazazi et al. [7], polymer flooding decreased the mobility ratio to less than 1 and resulted in 11% additional oil recovery after waterflooding. Literature shows that preferable conditions for successful polymer flooding can be achieved when the mobility ratio is less than or equal to 1 [8].

Several factors need to be considered when selecting or designing a polymer solution for a certain reservoir. One of the critical factors is the viscosity of the crude oil to be displaced by the polymer. To achieve a mobility ratio of less than 1, the viscosity of the polymer solution should be equal to or higher than the viscosity of the crude oil being displaced at reservoir conditions. The viscosity of a polymer solution can be adjusted by increasing the polymer concentration, lowering the salinity of the brine, and application of higher molecular weight polymers [9]. However, a too viscous polymer solution may result in operational problems such as high pressure in the near wellbore zone and injectivity issues. If the concentration of the polymer is very high, the project becomes economically unfeasible. The increasing molecular weight of the polymer can also cause injectivity problems and an increase in polymer adsorption and inaccessible pore volume [10].

Thus, the designed polymer solution must exhibit the desirable rheology at the reservoir conditions. The rheological behavior of polymer is dependent on several factors such as reservoir temperature, salinity, and microbial activity [11,12]. High salinity and the presence of divalent ions affect the negatively charged polymer chains, disrupt the molecular structure, and reduce the viscosity. It may also increase polymer adsorption on rock surfaces [13,14,15]. Hence, selecting and optimizing the type and concentration of the polymer for application in a specific field is critical for a successful mobility control project. 

Several fields are experiencing high water cuts as a result of waterflooding. A wide range of synthetic and biopolymers are available as EOR polymers for such oilfields [16]. However, hydrolyzed polyacrylamide (HPAM) polymer is widely used to reduce water production in most fields [17]. HPAM polymers are cost-effective, readily available, and can be modified and tailored according to target reservoir conditions [18]. Moreover, some modified HPAM polymers have high resistance against temperature and salinity and thus, can be used in fields with harsh reservoir conditions [19,20,21]. A successful HPAM polymer flooding application in a sandstone reservoir was reported in the Marmul oil field in south Oman [22]. Water cut dropped from 50% to 20% after polymer treatment, thereby improving well productivity and reducing the cost of surface facilities. The additional oil recovery by polymer flooding in this field was about 12% [8]. 

Polymer flooding was successfully implemented at Daqing Field, China [23]. It is a sandstone field with oil density ranging from 33 to 39° API, a depth of 900–1200 m, a permeability of 500–1000 mD, porosity ranging from 25 to 30%, and high wax content. Polymer flooding was initiated in this field in 1996 to control water cut and enhance oil production. Polymer injection proved successful as it recovered 12% additional oil from the field [23]. Zhong et al. [24] conducted experiments and field investigation of HPAM-based polymer flooding technology in the Daqing oilfield. Brine with a salinity of 395 ppm and divalent ions concentration of 36 ppm was used after filtration. Single-phase and two-phase flow characteristics measurements and oil displacement tests were conducted to evaluate the HPAM performance, and it was found that polymer flooding positively affected oil recovery from this reservoir. This study also showed that molecular weight and concentration of polymer are critical parameters affecting pore blockage, resistance factor, and formation damage. 

Successful implementation of polymer flooding to control water production was also reported in Aishwarya Field, Rajasthan, India [25]. The oil produced in the Aishwariya field has high wax content and a viscosity of 10–30 cp in-situ. Screening analysis recommended the application of polymer flooding for the field. Polymer flooding was implemented in two stages. In the first stage, the performance of the polymer was tested in the field by converting 2 of the producers into polymer injectors. As a result, a significant reduction in water cut from 57% to 29% and an increase in oil rate from 1700 BPD to 2100 BPD were observed at producers in the vicinity of the injectors. Pressure support increased viscosity around the wellbores, and stabilized water–oil-ratio was also observed in nearby wells. In the second stage, full-field polymer injection at Lower Fatehgarh formation was designed and implemented. As a result, the oil rate increased from 7000 bpd to 10,700 bpd and the water cut decreased from 84% to 81%. In some wells, water production was significantly reduced with water cut as low as 45% [25].

Polymer flooding at the Bhagyam Field, Rajasthan, India was also effectively executed. The oil produced in the Bhagyam sandstone field has a viscosity of 15–20 cp [26]. The permeability ranges between 1 to 10 Darcy while porosity is in the range of 25–30%. Considerable reduction in water cut was observed in all the producers in the field with an increase of 5000 BPD in field oil production rate and 38% decrease in field WOR in response to polymer flooding [27]. Al Khalata sandstone reservoir in the Sultanate of Oman is another example of a successful polymer flooding project. As a result of polymer flooding in the reservoir, the water cut dropped by 2–30% and the oil production increased by about 25% [28]. 

Field A in Kazakhstan faces a similar problem of high water cut. Waterflooding for pressure maintenance was started in the field at the early stage of field development. After years of waterflooding, water production in all the wells is very high, with field-wide water cut reaching 90%. The fluid and rock properties of Field A are analogous to the fields discussed above. For example, the oil is waxy, the lithology is sandstone, and permeability and porosity are in the range of 100–500 md and 24–27%, respectively. The crude oil in Field A has a density of around 0.80 g/cc while the viscosity of the oil is 4.5 cp at a reservoir temperature of 63 °C. The oil also contains 13–15 wt% asphaltenes and 20–28 wt% paraffin content. The formation water of Field A has a high salinity of 120,000 ppm with a high concentration of monovalent and divalent ions. The success of polymer flooding in fields similar to the sandstone oilfield in Kazakhstan implies that the implementation of the polymer EOR method can enhance oil production by controlling water-cut in Field A.

Since Field A fulfills the necessary screening criteria to be a candidate for polymer flooding, a systematic polymer screening study is required for successful polymer injection in Field A. This paper presents an evaluation of different HPAM polymers to select the optimum one for Field A based on viscosification, thermal stability, adsorption tendency, resistance to bacterial activity, and tolerance to oxygen. The main target of this research is to follow a robust experimental approach to study the polymer rheology, stability, and adsorption as the main criteria for the selection of the most efficient polymer before evaluating its performance in the porous media. The effect of the condition of make-up brine on the aforementioned parameters is also investigated during the screening process.

## 2. Materials and Methods 

### 2.1. Polymer Solutions

The make-up brine to be evaluated for polymer solution preparation is the Caspian Seawater (CSW) as it is available to be used for the target field. The ionic composition of the CSW is presented in Table 1. Since water samples from the fields are not readily available to be used in various research studies, synthetic brines are widely used in lab tests. A similar approach is used in this study to first prepare all polymer samples in synthetic seawater for the initial screening of polymers. Based on a preliminary study and the application of HPAM polymers in similar fields, four types of HPAM polymers are selected for the initial screening phase. Table 2 shows some of the physical properties of each polymer. The polymer solutions are prepared at concentrations of 500, 1000, 1500, 2000, and 3000 ppm.

Since the objective of this study is to screen the optimum brine–polymer combination, the condition of the make-up brine is one of the most critical parameters. Generally, formation brine samples, seawater, or a designed brine are used to prepare polymer solutions for a polymer flooding project. For example, Chen et al. [29] used processed water for the polymer screening laboratory experiments. Zhangaliyev et al. [30] prepared a synthetic brine based on the formation field brine composition. The same approach was followed by Ulasbek et al. [31]. Wei et al. [32] also prepared synthetic brines based on the formation water composition. 

To study the effect of different factors such as the presence of non-ionic components including bacteria and the effect of oxygen on the performance of polymer solution, both synthetic brine and seawater are used in this study for a reliable screening criterion. The presence of oxygen in the solution brine affects the stability and the rheology of the polymer. Imanbayev et al. [33] showed that it is critical to consider the oxygen content in the make-up brine. They tried to decrease oxygen content to undetectable levels by using chemical or gas treatment. Jolene et al. [34] prepared synthetic brine in the glove box to keep the oxygen content below 1 ppb. Similarly, Seright and Skjevrak [35] prepared anaerobic synthetic brine in an anaerobic chamber to remove oxygen. In their study, the brine was circulated through a palladium catalyst and a desiccant with anaerobic gas (10–15% hydrogen and 85–90% nitrogen) to convert free oxygen, and hydrogen to water and remove them. Purging with nitrogen is another method to remove oxygen from the solution. 

In this study, several types of brines were prepared to investigate the effect of parameters such as oxygen and non-ionic components on the screening process. In the first step, synthetic seawater was prepared by adding salts (NaCl, KCl, MgCl_2_, CaCl_2_, NaSO_4_, Na_2_CO_3_, and NaHCO_3_ supplied by Honeywell) to the distilled water according to CSW composition and mixing thoroughly with a magnetic stirrer. The objective of using synthetic water was to eliminate the effect of non-ionic materials present in the CSW and to obtain the rheological data in an aerobic condition. This stage was designed to select the polymers exhibiting the desired viscosity, the appropriate shear-thinning behavior, and maximum thermal stability for further testing and screening. In the next phase, the effect of non-ionic components was studied by preparing polymer solutions in the collected CSW samples. CSW was filtered using a Whatman filter paper of 30 μm pore size to remove any solid particles. In the final stage of the screening, the effect of oxygen on polymer viscosity and thermal stability was studied by preparing polymer solutions at an anaerobic condition. Anaerobic samples were prepared by glove box and nitrogen purging method in which a thin steel tube was placed at the bottom of the water sample and pure nitrogen gas was injected from the bottom. The beaker was covered with a parafilm with two holes for nitrogen inflow and oxygen outflow. 

To prepare a stable polymer solution, different approaches are proposed in the literature. In our study, polymer solutions were prepared according to API RP 63 standard using a magnetic stirrer [36]. A precisely weighed amount of solid dry polymer was added to the selected brine to obtain a polymer solution at a desired concentration. A 70% vortex was maintained while adding dry polymer to the aqueous solution. The solution was then left overnight, stirring at a low speed of 150 rpm. This procedure ensures complete dissolution and hydration of polymer molecules in the make-up brine and also eliminates the chance of fisheyes formation.

### 2.2. Rock Samples

To analyze the adsorption of different brine–polymer combinations on reservoir rock, sandstone rock samples were crushed and mixed with different polymer solutions for static adsorption analysis. The main constituent of the rock was quartz with some percentage of clay particles as well [37].

### 2.3. Screening Methodology

An acceptable polymer solution to reduce the mobility in a reservoir should be stable at the high-temperature reservoir condition for a long time, faces low degradation and adsorption, and flows at the target viscosity to change the mobility ratio to a favorable condition. Hence, the screening methodology in this study was based on stability tests, thermal degradation studies, and static adsorption tests. Based on the results of these studies at different conditions such as aerobic/anaerobic and synthetic brine/seawater, the best polymer–brine combination was screened. The following sections describe the experiments conducted for this screening study.

### 2.4. Rheology Analysis

The objective of this phase was to investigate the effect of polymer concentration, shear rate, aging time, and type of brine on the viscosity of polymer solutions. The target viscosity of the polymer was set as 5 cp at a reservoir temperature of 63 °C at a typical field shear rate of 10 s^−1^. As already mentioned, the crude oil viscosity in the target field is around 4.5 cp, and thus, target polymer viscosity of 5 cp was chosen to keep the mobility ratio in the reservoir equal to or greater than 1. Polymer solutions of the four HPAM polymers were prepared in different brines for viscosity analysis. The Modular Compact Rheometer (MCR 302) sourced from Anton Paar, Graz, Austria, was used to conduct the bulk scale rheological investigation. The shear viscosity was measured at 63 °C using a double wall concentric cylinder geometry for polymer solutions in the range of 500 ppm to 3000 ppm. Viscosity was measured by filling 50 mL of the sample in a rheometer cylinder and performing a shear scan over shear rates in the range of 1–100 s^−1^.

### 2.5. Thermal Stability Study

One of the characteristics of an optimum brine–polymer combination is that the injected polymer should be stable for at least 2 weeks inside the reservoir to reduce water mobility and thus the water cut. Hence, this point was considered as one of the screening criteria to select the best polymer-brine blend. The long-term thermal stability test was conducted to evaluate the ability of polymer solutions to maintain the viscosity at reservoir temperature for a longer period (120 days). Two levels of polymer concentration, a higher one and a lower one, were chosen for the testing of each polymer to account for economic and technological expediency. For this purpose, a procedure similar to the one reported by Sandengen et al. [38] was followed. The polymer solutions prepared at an optimum concentration as determined from the bulk rheological study were poured into 50 mL vials and placed into the oven at the reservoir temperature of 63 °C. The test tubes were sealed to prevent any evaporation and to ensure anaerobic conditions. The viscosity of samples was measured at regular intervals every week to develop the thermal degradation curve and evaluate the thermal stability of the polymer. As the polymer degradation is usually higher during the first month of the experiment, the frequency of the viscosity measurement was kept higher during the first 30 days. Equation (2) was used to calculate thermal degradation after a certain period of aging at reservoir temperature.

(2) Thermal Degradation,%=μ0−μtμ0×100

where *μ*_0_ and *μ_t_* are polymer solution viscosities at 0 days and *t* days of the thermal stability test, respectively.

### 2.6. Static Adsorption Test

The tendency of the polymer to adsorb on the rock surface negatively affects the efficiency of a polymer flooding project by retarding the polymer front advance [39]. Therefore, the screening of the optimum brine–polymer combination requires a detailed study of the adsorption behavior of polymers [9]. For this purpose, the polymers short-listed based on the rheology and thermal stability study results were further evaluated by conducting static adsorption tests on sandstone crushed rock samples at different concentrations, residence times, and polymer solution to rock mass ratio. Samples with concentrations of 1000, 1500, and 2500 ppm were prepared for each polymer and mixed with the crushed sandstone at liquid-to-solid ratios ranging between 2–100. The samples were kept in a roller oven to enable uniform mixing for one week. The samples were then transferred to 10 mL vials and absorbance of each sample was measured using an Evolution 300 UV-Vis spectrophotometer by Thermo Scientific sourced from Waltham, Massachusetts, United States, after 3, 12, 24, and 36 h residence time. A calibration curve was developed for each polymer to convert the absorbance values obtained from UV into change in concentration. Static adsorption was then calculated using Equation (3):

(3) $$A_{p}=\left(C_{i}-C\right)\times LSR$$

where *A_p_* is the static adsorption density of polymer (*μ_g_*/g of rock), *C_i_* is the initial polymer concentration (ppm), *C* is the polymer concentration after the polymer is adsorbed on the rock (ppm), and *LSR* is the liquid-to-solid ratio.

## 3. Results and Discussion

This section presents the results of different phases of the screening study to select the best polymer–brine design for the target field A.

### 3.1. Rheological Characterization

**Figure 1:**
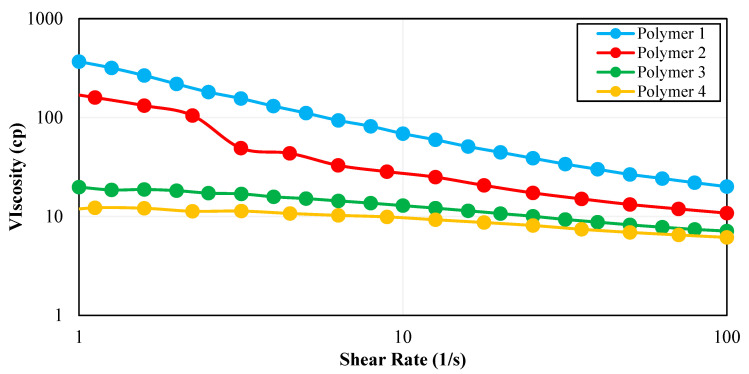
Viscosity profiles of four polymers at 63 °C for a polymer concentration of 2000 ppm.

**Figure 2:**
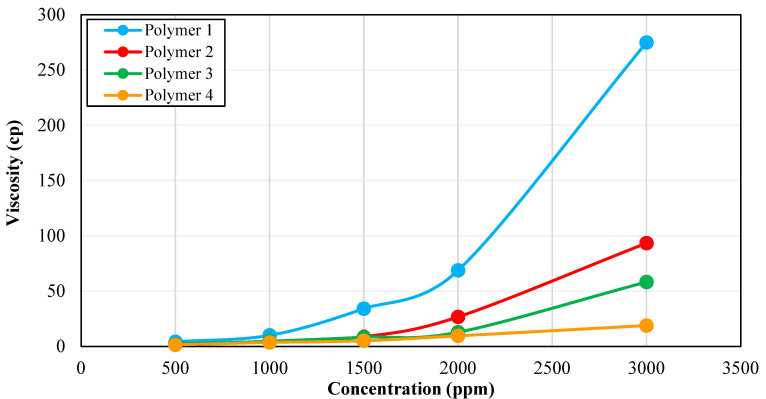
Rheology results of four HPAM-based polymers evaluated at a shear rate of 10 s^−1^.

All four polymer solutions were prepared in synthetic seawater to remove the effect of non-ionic materials. Additionally, these solutions were prepared under aerobic conditions. It was observed that all polymers followed the shear thinning behavior (Figure 1) which is an important consideration for field applications of polymers [40]. At a high shear rate, which is experienced by the fluid in the tubing during injection, the polymer should have lower viscosity to minimize injectivity problems. Conversely, at a low shear rate, which happens in the reservoir, the polymer should have higher viscosity to achieve a lower mobility ratio and more piston-like movement. Figure 2 shows the rheology test results for all four polymers as a function of concentration. 

As expected, the polymer solutions of higher concentrations exhibited higher viscosities. The viscosities for all samples were compared at a shear rate of 10 s^−1^ which is considered a representative shear rate during the fluid flow in the reservoir. Although Polymer 1 showed the highest viscosities in the range of 10–275 cp among the four polymers, the Polymer 1 viscosity was much higher than the target viscosity of 5 cp and it behaved more like a gel with pronounced viscosifying power. Hence, Polymer 1 was excluded from further screening stages due to possible injectivity problems in the field.

### 3.2. Long-Term Thermal Stability 

**Figure 3:**
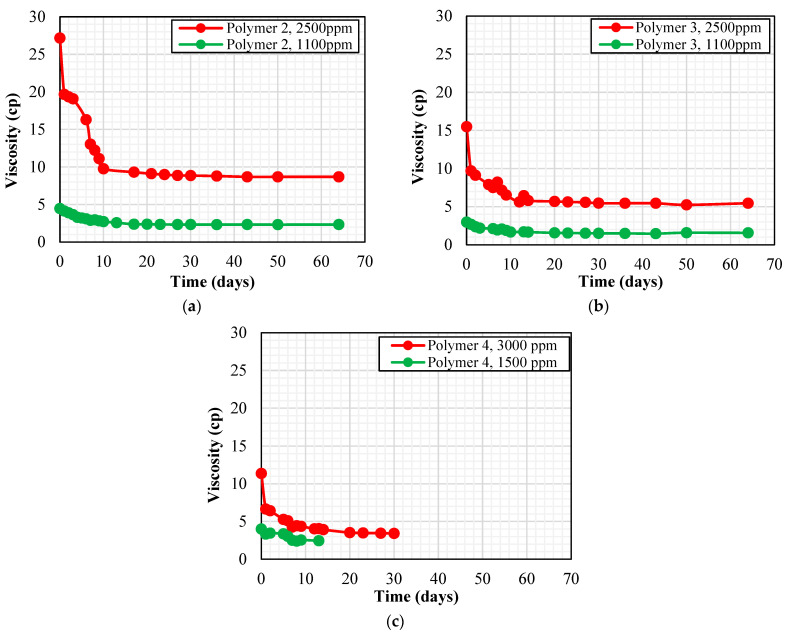
Thermal stability results at a shear rate of 10 s^−1^ for (**a**) Polymer 2, (**b**) Polymer 3, and (**c**) Polymer 4.

Our preliminary studies showed that at the shear rate of 10 s^−1^, polymer solution viscosity should be about 5 cp to keep the mobility ratio below one. Hence, the target was to select polymers and appropriate concentrations to maintain this value at reservoir conditions for a long time (at least for two weeks). Several high and low polymer concentrations were analyzed at 63 °C for two months. The high and low concentrations were 2500 ppm and 1100 ppm, respectively, for both Polymer 2 and Polymer 3. Since Polymer 4 exhibited lower viscosity compared to other polymers at all concentrations during the preliminary rheology test, higher concentrations for Polymer 4 (3000 ppm and 1500 ppm) were chosen for thermal stability analysis. Figure 3 shows some of the thermal stability test results for polymers 2, 3, and 4. It can be observed from Figure 3 that Polymer 4 was unstable at seawater salinity and the solution viscosity quickly reduced below the 5 cp target viscosity, even at a high concentration of 3000 ppm. As a result, polymer consumption to reach the target viscosity would be higher in the case of Polymer 4, negatively affecting the project’s economics. Hence, the thermal stability test for Polymer 4 was discontinued and Polymer 4 was excluded as it was not effective to reduce the mobility ratio. During the two months of thermal stability study, both Polymer 2 and Polymer 3 exhibited favorable viscosity at 2500 ppm concentration. As a result, these polymers were selected for the next stages of screening which comprised comparing the effect of non-ionic materials, the effect of oxygen, and static adsorption results.

### 3.3. Effect of Non-Ionic Materials

The water coming from natural sources such as seawater generally contains non-ionic materials in addition to salts. These non-ionic materials include sediments of rocks, organic compounds such as phthalates, dissolved organic carbon, and non-ionic surfactants [41,42,43], and microorganisms, mainly bacteria [44,45]. The sediments can be removed from natural water samples by the process of filtration. The organic materials and bacteria, however, can only be removed by specific chemical treatment processes. 

**Figure 4:**
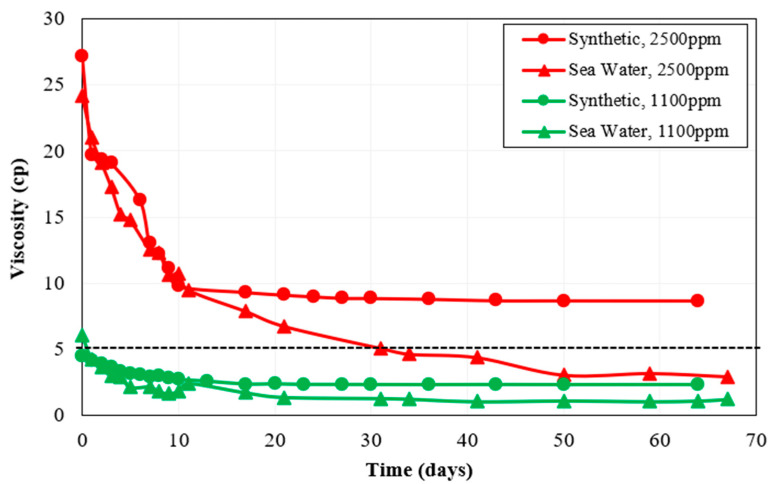
Effect of non-ionic material on Polymer 2 thermal stability at a shear rate of 10 s^−1^.

According to the work conducted by Nazina et al. [44], the Caspian seawater contains temperature-resistant bacteria that are capable of sulfate reduction. Moreover, the presence of sulfate-reducing bacteria in Caspian Seawater is also supported by a high content of H_2_S in Caspian Seawater reported by Ivanov et al. [46], as the authors performed a detailed biogeochemical analysis of the water samples from a Caspian Sea water column. In this context, after choosing the best polymers based on rheology and long-term thermal stability analysis, the next step was to select the best brine–polymer combination among Polymer 2 and Polymer 3 based on the performance of each polymer in the presence of bacteria in the make-up brine. For this purpose, polymer solutions were prepared in CSW. The primary difference between polymer solutions prepared synthetically and with seawater was due to the presence of bacteria in CSW. Polymer 2 and Polymer 3 samples prepared with CSW as make-up brine were then tested for long-term aerobic thermal stability. Figure 4 shows Polymer 2 thermal stability results for synthetic brine and seawater. 

It can be seen from Figure 4 that the viscosity of Polymer 2 rapidly decreased in seawater during the thermal stability test, indicating higher degradation of Polymer 2 due to the activity of thermophilic bacteria present in CSW. Sulfate-reducing bacteria can cause hydrolysis of the HPAM side chain amide group to the carboxyl group which results in the alteration of polymer functional units. As a result, long polymer molecular chains break and the viscosity of the polymer solution is reduced by the action of sulfate-reducing bacteria [47]. Sulfate-reducing bacteria also produce sulfide ions as their metabolic product which have been shown to negatively affect the viscosity of HPAM solutions by decreasing the polymer hydrodynamic radius and destroying the intra-inter molecular structure of the polymer [48]. Owing to these factors, the thermophilic sulfate-reducing bacteria present in CSW caused higher degradation of Polymer 2 as they were active at a high temperature of 63 °C. Over one month, Polymer 2 lost around 79% of its viscosity at both high and low concentrations. 

**Figure 5:**
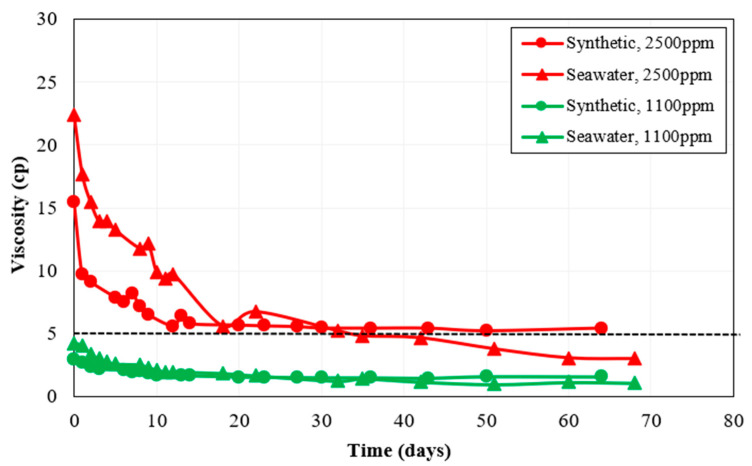
Effect of non-ionic material on Polymer 3 thermal stability at a shear rate of 10 s^−1^.

On the other hand, the Polymer 3 solution prepared in CSW showed higher viscosity values at both concentrations of 1100 ppm and 2500 ppm for the first month of the thermal stability test, as can be seen in Figure 5. However, after a one-month period, the viscosity curve of Polymer 3 prepared with CSW went below the viscosity curve of Polymer 3 prepared synthetically. These results indicate a significant difference between the properties of Polymer 2 and Polymer 3. In the case of Polymer 3, the presence of bacteria in CSW caused an increase in solution viscosity. The viscosity of 2500 ppm Polymer 3 prepared in CSW was 74% higher compared to the same polymer prepared in synthetic seawater after two weeks. This increase in viscosity can be attributed to increased hydrolysis of polymer molecules by bacteria, generating more carboxyl ions on the polymer backbone and thereby causing more repulsion between molecular chains. As a result, the viscosity of the Polymer 3 solution increased in CSW. Unlike Polymer 2, Polymer 3 showed almost similar viscosity in both types of brines after one-month aging at 63 °C. 

Once the polymer reaches an optimum degree of hydrolysis, further hydrolysis by bacterial activity can cause a reduction in solution viscosity by converting amide groups to acrylate groups and degrading the molecular chains [49]. Thus, increased hydrolysis above the optimum value can be the reason for the reduction in Polymer 3 viscosity in CSW after 1 month. The viscosity of Polymer 3 in CSW after 1 month was only 4% below that of the same polymer in synthetic brine, whereas the viscosity of Polymer 2 in CSW was around 43% less than its viscosity in synthetic seawater. Overall, the results obtained in Figure 5 show that Polymer 3 is more stable in the presence of bacteria in make-up brine compared to Polymer 2. 

### 3.4. Effect of Oxygen

**Figure 6:**
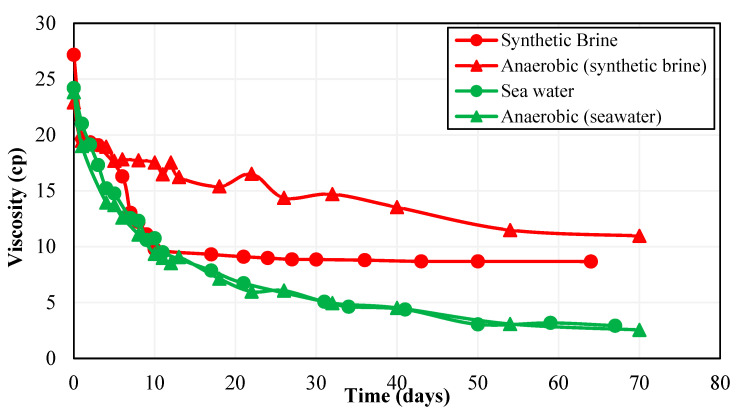
Effect of oxygen on Polymer 2 thermal stability at a shear rate of 10 s^−1^.

Another important factor influencing polymer performance assessed during this study is the effect of oxygen. The thermal stability test was repeated for Polymer 2 and Polymer 3 prepared in synthetic seawater and CSW under anaerobic conditions. Polymer solutions were deoxygenated using the nitrogen purging method. Figure 6 shows the comparison of the thermal stability of Polymer 2 at 2500 ppm concentration in the presence and absence of oxygen. It can be seen that the anaerobic conditions positively affected the performance of Polymer 2 prepared in synthetic seawater as the solution viscosity after one month of aging was almost 2 times higher in the absence of oxygen. Moreover, the thermal degradation also reduced from 69% in aerobic synthetic brine to 36% in an anaerobic environment. Higher thermal degradation of the polymer in presence of oxygen can be attributed to oxidization reactions generating free radicals such as sulfide in the solution [50].

Sulfide ion has been reported to degenerate HPAM polymer acrylamide chains and causes a reduction in the hydrodynamic radius of the polymer, thereby lowering polymer solution viscosity [48]. On the contrary, there is a negligible effect of oxygen on the thermal stability of CSW-based polymer solution, displaying approximately similar behavior over a one-month aging period. Researchers studying the oxygen content in various water sources have reported a lower oxygen concentration in seawater compared to fresh water at the same partial pressure of oxygen [51]. It can be due to the fact that CSW samples already contained low oxygen concentration and thus the oxygen content after nitrogen purging did not change significantly. Consequently, the presence of oxygen in seawater did not affect the thermal stability behavior of Polymer 2. 

**Figure 7:**
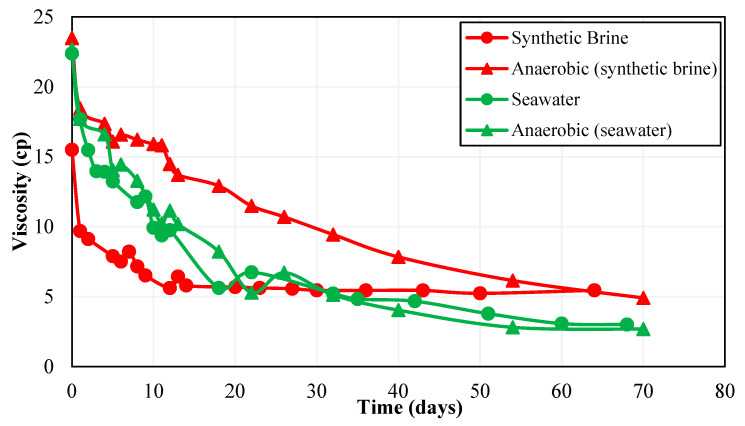
Effect of oxygen on Polymer 3 thermal stability at a shear rate of 10 s^−1^.

Similar results were observed for Polymer 3 as can be seen in Figure 7. The anaerobic conditions helped improve the thermal stability of the polymer in synthetic seawater samples as the polymer viscosity was ~46% higher in an anaerobic sample compared to an aerobic sample after the one-month aging period at 63 °C. The higher reduction in polymer viscosity can again be attributed to the oxidation of the polymer molecules in the aerobic medium. Observations similar to Polymer 2 can be made from the results of Polymer 3 that the presence of oxygen had a minimum effect on the viscosity profile of polymer solutions prepared with CSW. Hence, the results of this stage indicated that both polymers were unaffected by the presence of oxygen in CSW, seawater without deoxygenation can be used as make-up brine for polymer flooding in Field A.

### 3.5. Static Adsorption Results

**Figure 8:**
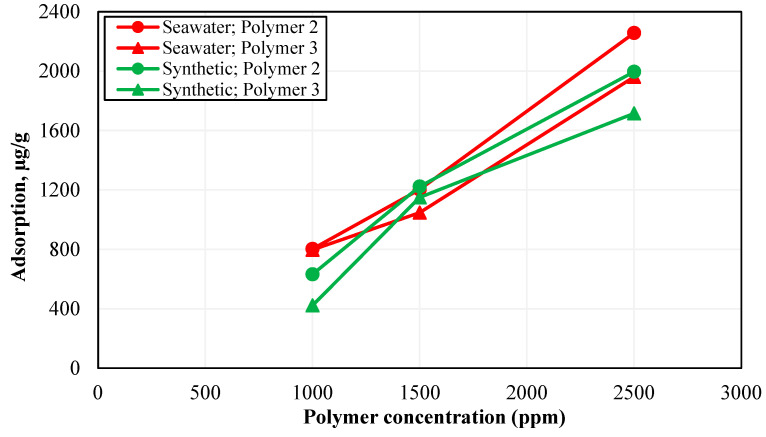
Static adsorption results for Polymer 2 and Polymer 3 in synthetic brine and seawater.

After screening the polymers based on long-term thermal stability, the effect of non-ionic content, and sensitivity to the presence of oxygen, the final stage was to select the best polymer based on its static adsorption tendency. The criterion was to select the polymer with the lowest static adsorption values. Considering the results of some static adsorption studies conducted on sandstone material, the adsorption cut-off was set as 2000 μg/g of rock [9]. Figure 8 shows the static adsorption as a function of concentration for both Polymer 2 and Polymer 3 after 36 h of settling time at a liquid-to-solid ratio of 10. Since anaerobic conditions showed a negligible effect on the performance of both polymers in CSW, the static adsorption test was conducted for synthetic brine and seawater in aerobic conditions. The general trend was that static adsorption increased with increasing polymer concentration, due to the availability of a larger number of polymer molecules.

The polymer adsorption values in the range of 1700 to 2500 μg/g of rock were obtained for different brine–polymer combinations at 2500 ppm polymer concentration. Similar static adsorption results have been reported by Al-Hajri et al. (2020) where static adsorption for various HPAM-based polymers after 24 h of residence time with crushed sandstone was in the range of 1300 to 3800 μg/g of rock at 1000 ppm polymer concentration and a liquid-to-solid ratio of 13. It can be seen from Figure 8 that Polymer 3 has lower static adsorption than Polymer 2 at all concentrations in both synthetic brine and seawater. The static adsorption of Polymer 3 at a concentration of 2500 ppm was 13–14% less compared to that of Polymer 2 in both make-up brines. The lower static adsorption of Polymer 3 can be explained by higher stability and repulsion between its molecular chains, resulting in a smaller number of molecules required to satisfy the adsorption capacity of sandstone grains.

**Figure 9:**
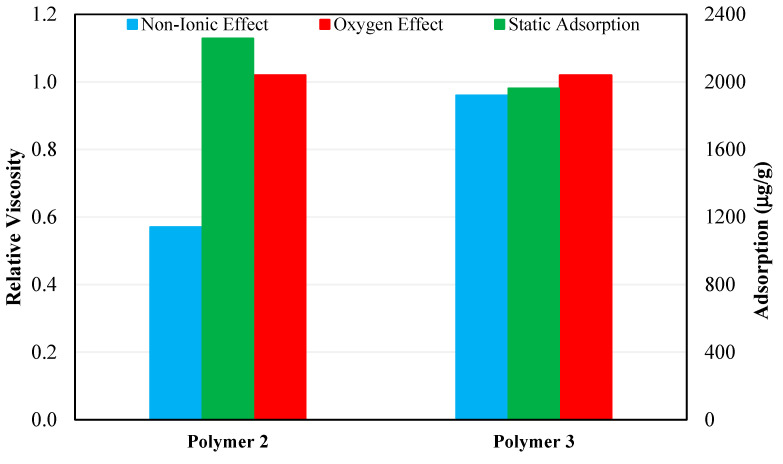
Comparison of Polymer 2 and Polymer 3 in terms of relative viscosity and adsorption.

Another observation regarding the adsorption behavior of polymers in synthetic brine and seawater from Figure 8 is that both polymers showed around 13–14% higher adsorption in CSW compared to synthetic seawater. This higher adsorption can be due to the degradation of polymer chains caused by bacterial activity in CSW, resulting in a decreased hydrodynamic radius and increased adsorption of polymer on the rock. Figure 9 compares the performance of Polymer 2 and Polymer 3 prepared in CSW in terms of resistance to non-ionic materials and oxygen and the tendency to adsorb on the rock surface. The relative viscosity in Figure 9 was obtained using Equation (4):

(4) $$Relative\;Viscosity={\left(\frac{\mu_{R}}{\mu_{S}}\right)}_{One-month\;aging\;time}$$

where *μ_R_* is the viscosity of 2500 ppm polymer solution prepared in CSW and *μ_S_* is the polymer solution viscosity prepared in synthetic seawater. Based on long-term thermal stability, oxygen effect, and the effect of non-ionic material, the 2500 ppm concentration depicted the best performance as it maintained the target viscosity of 5 cp over longer durations and thus, was used for comparison purposes.

From the comparison shown in Figure 9, it can be concluded that Polymer 3 is more compatible and has a higher tolerance for non-ionic material, such as bacteria in seawater, than Polymer 2. The compatibility of polymer with seawater is an important criterion for screening as the most suitable and economically feasible source of make-up brine for polymer flooding in Field A is CSW.

Table 3 summarizes the screening criteria followed in this study and the evaluation of each polymer against these screening criteria. Polymer 1 was excluded because of its gelling tendency and possible injectivity issues in the field which can be due to its higher molecular weight and a very high degree of hydrolysis compared to other polymer systems. Polymer 4 was discarded owing to its lower thermal resistance. Polymer 2 showed a higher static adsorption tendency due to its higher molecular weight compared to Polymer 3. Overall, Polymer 3 has depicted superior performance concerning each screening criterion and is recommended to be used during pilot testing on Field A.

## 4. Conclusions

In this study, four HPAM polymer samples were evaluated for potential polymer flooding application for one sandstone oil reservoir in Kazakhstan. The screening criteria comprised testing the polymers for rheology, thermal stability, tolerance to bacteria and oxygen presence in make-up brine, and static adsorption analysis.

The results obtained in this study demonstrated critical parameters to be considered while screening polymers for a certain oilfield. The ability of a polymer to withstand high reservoir temperature and maintain target viscosity for an extended duration in the reservoir was the first criterion for polymer selection for Field A. The effect of non-ionic materials such as bacteria present in the potential make-up brine was another important aspect evaluated in this screening study. For a successful and optimum design of a polymer flood project, the chosen polymer must be tolerant to non-ionic contents, and more importantly, it should have minimum degradation in response to bacterial activity. Likewise, the resistance of polymer to dissolved oxygen in make-up brine was another parameter to be considered in evaluating the polymers for Field A. Dissolved oxygen can cause oxidation of polymer molecules and result in a decrease of the hydrodynamic volume of the polymer. Finally, the adsorption potential of a polymer considerably affects the technical and economic feasibility of the polymer flooding project. Thus, the final step in this screening study was to test the available polymers for static adsorption tendency.

By considering the above-mentioned parameters, one polymer among the four available polymers was selected as it maintained the target viscosity of 5 cp after two weeks of aging at reservoir temperature during the thermal stability study. This polymer also showed the highest stability in presence of non-ionic materials particularly bacteria in CSW as its viscosity after one-month aging was almost the same as in the synthetic bacteria-free seawater sample. The selected polymer also resulted in 13–14% lower adsorption on sandstone crushed rock compared to other polymers tested. Therefore, this polymer passed the screening criteria set in the study for the specific reservoir and it can be recommended for pilot testing in Field A.

## Figures and Tables

**Table 1:** Ionic composition of Caspian seawater.

Ion	Concentration (ppm)
Na^+^	3513.1
Ca^2+^	400.8
Mg^2+^	790.4
Cl^−^	6026
SO_4_^2−^	3138
HCO^3−^	256.2
K^+^	87.6
CO_3_^2−^	36

**Table 2:** Physical properties of the four polymers used in the study.

Parameters	Units	Polymer 1	Polymer 2	Polymer 3	Polymer 4
Molecular weight	×10^6^, Dalton	12.2	11.1	7.6	8.4
Intrinsic viscosity	dL/g (deciliter/g)	18.8	17.3	12.9	14
Degree of hydrolysis	%	19.7	1.2	6.4	7.4

**Table 3:** Screening criterion for the selection of optimum brine-polymer combination for Field A.

Test	Criterion	Polymer 1	Polymer 2	Polymer 3	Polymer 4	Remarks
Rheology	≥5 cp viscosity, No gelling	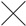	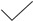	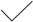	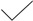	Polymer 1 appeared more like a gel and was discarded.
Thermal Stability	≥5 cp viscosity for at least two weeks aging at 63 °C	-	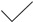	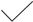	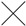	Polymer 4 was eliminated as it did not meet the set criterion.
Non-Ionic Material (Bacteria)	Little to negligible effect on thermal stability over a month	-	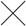	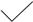	-	Polymer 2 showed higher thermal degradation in presence of bacteria.
Oxygen Content	Little to negligible effect on thermal stability over a month	-	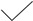	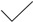	-	Both polymers showed a negligible effect of oxygen present in CSW.
Static Adsorption	A_p_ ≤ 2000 μg/g	-	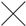	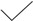	-	Static adsorption for Polymer 2 was higher.

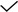
 Meets the criterion; 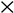 Does not meet the criterion.

## Data Availability

Not applicable.
